# Src and ROCK Kinases Differentially Regulate Mineralization of Human Osteosarcoma Saos-2 Cells

**DOI:** 10.3390/ijms20122872

**Published:** 2019-06-12

**Authors:** Agnieszka Strzelecka-Kiliszek, Marta Romiszewska, Lukasz Bozycki, Saida Mebarek, Joanna Bandorowicz-Pikula, Rene Buchet, Slawomir Pikula

**Affiliations:** 1 Laboratory of Biochemistry of Lipids, Nencki Institute of Experimental Biology, Polish Academy of Sciences, 3 Pasteur Str., 02-093 Warsaw, Poland; a.strzelecka-kiliszek@nencki.gov.pl (A.S.-K.); m.romiszewska@nencki.gov.pl (M.R.); l.bozycki@nencki.gov.pl (L.B.); 2 Université de Lyon, CEDEX 69622 Villeurbanne, France; saida.mebarek@univ-lyon1.fr (S.M.); rene.buchet@univ-lyon1.fr (R.B.); 3 Université Lyon 1, CEDEX 69622 Villeurbanne, France; 4 NSA de Lyon, CEDEX 69621 Villeurbanne, France; 5 CPE Lyon, CEDEX 69616 Villeurbanne, France; 6 ICBMS CNRS UMR 5246, CEDEX 69622 Villeurbanne, France; 7 Laboratory of Cellular Metabolism, Nencki Institute of Experimental Biology, Polish Academy of Sciences, 3 Pasteur Str., 02-093 Warsaw, Poland; j.bandorowicz-pikula@nencki.gov.pl

**Keywords:** Src kinase, ROCK, annexin A6, mineralization, matrix vesicles, Saos-2 cells

## Abstract

Osteoblasts initiate bone mineralization by releasing matrix vesicles (MVs) into the extracellular matrix (ECM). MVs promote the nucleation process of apatite formation from Ca^2+^ and P_i_ in their lumen and bud from the microvilli of osteoblasts during bone development. Tissue non-specific alkaline phosphatase (TNAP) as well as annexins (among them, AnxA6) are abundant proteins in MVs that are engaged in mineralization. In addition, sarcoma proto-oncogene tyrosine-protein (Src) kinase and Rho-associated coiled-coil (ROCK) kinases, which are involved in vesicular transport, may also regulate the mineralization process. Upon stimulation in osteogenic medium containing 50 μg/mL of ascorbic acid (AA) and 7.5 mM of β-glycerophosphate (β-GP), human osteosarcoma Saos-2 cells initiated mineralization, as evidenced by Alizarin Red-S (AR-S) staining, TNAP activity, and the partial translocation of AnxA6 from cytoplasm to the plasma membrane. The addition of 4-amino-5-(4-chlorophenyl)-7-(t-butyl)pyrazolo [3,4-d] pyrimidine (PP2), which is an inhibitor of Src kinase, significantly inhibited the mineralization process when evaluated by the above criteria. In contrast, the addition of (R)-(+)-trans-4-(1-aminoethyl)-N-(4-pyridyl) cyclohexane carboxamide hydrochloride (Y-27632), which is an inhibitor of ROCK kinase, did not affect significantly the mineralization induced in stimulated Saos-2 cells as denoted by AR-S and TNAP activity. In conclusion, mineralization by human osteosarcoma Saos-2 cells seems to be differently regulated by Src and ROCK kinases.

## 1. Introduction

Sarcoma proto-oncogene tyrosine-protein (Src) kinase [1,2] as well as Rho-associated coiled-coil (ROCK) kinases [3] may regulate actomyosin skeleton dynamics that prompt diverse cellular activities such as proliferation, differentiation, motility, adhesion, transcription, survival, angiogenesis, endocytosis, exocytosis, and cytokinesis. However, there is very little experimental evidence about their direct or indirect links with the mineralization process. Since interactions of growth plate chondrocytes with extracellular matrix (ECM) proteins regulate cell behavior, primary chondrocyte adhesion and spreading dynamics on fibronectin (FN) and bone sialoprotein (BSP) were compared [4]. The inhibition of ROCK activity with (R)-(+)-trans-4-(1-aminoethyl)-N-(4-pyridyl) cyclohexane carboxamide hydrochloride (Y-27632) increased cell spreading on BSP and membrane protrusions on FN, but did not affect cell adhesion, suggesting that the Rho/ROCK pathway inhibits chondrocyte spreading on BSP [4]. Recent discoveries in the field of bone biology indicate the participation of the Src family of tyrosine kinases and the Rho family of small GTPases in mineral formation [5]. Recently, we have established the possible involvement of the RhoA/ROCK pathway during matrix vesicle release [6]. 

Skeletal cells initiate mineralization by releasing submicroscopic matrix vesicles (MVs) [7]. MVs promote the formation of apatite in their lumen, bud from the microvilli of osteoblasts or chondrocytes during bone formation and development, and finally expel the preformed crystals to the ECM [8,9,10]. Both annexins and tissue non-specific alkaline phosphatase (TNAP), which are present in relatively high concentrations as compared to other types of extracellular vesicles, have collagen-binding capacity, which is a property that may help align MVs along collagen fibers to promote the propagation of mineralization onto the ECM scaffold [7].

Experimental evidence suggested that annexins, as calcium-binding and phospholipid-binding proteins, are engaged in the calcium homeostasis of mineralizing cells and the influx of Ca^2+^ to MVs [11,12,13]. The large variety of annexins in MVs indicates that they have different functions during the mineralization process [14]. For example, AnxA2 and AnxA6, which are expressed on the cell surface, served as receptors for adhesion to immobilized fetuin-A [15]. AnxA5 bound to the outer membrane of MVs interacted with ECM proteins such as collagen type II [11,16,17]. AnxA5 at the inner side of MVs initiated the nucleation process [16]. AnxA2, AnxA5, and AnxA6 mediated Ca^2+^ ion influx into MVs [12,18]. Acidic pH induced the formation of the AnxA6 ion channel [19]. However, AnxA2, AnxA5, and AnxA6 are not typical ion channels, and it is probable that they facilitate calcium binding rather than calcium transport. AnxA6 was found to be almost non-extractable from MVs by ethylene glycol-bis(β-aminoethyl ether)-N,N,N′,N′-tetraacetic acid (EGTA). Moreover, our report provided evidence of the presence of annexin-binding S100 proteins in MVs derived from mineralizing cells. We observed that upon mineralization, S100A10 and S100A6—but not S100A11—translocated to the MVs of Saos-2 cells in selective way [20].

We speculated that submembraneous cytoskeletal proteins, which are substrates for Src kinases and Rho small GTPases, such as non-muscle myosin II (MII), serve as platforms of vesicular transport. In addition, these proteins may control different steps of receptor transport and sorting to different organelles in the cell [21]. Also, the kinases and proteins of the cellular adhesion system, such as Focal Adhesion Kinase (FAK), vinculin, and paxillin, may be involved in the signaling pathway depended on ROCK and may connect plasma membrane integrins with the actin cytoskeleton in cell-to-cell as well as cell-to-extracellular matrix contact sites [22].

Understanding the mechanisms of cross-talk between the Src kinases and Rho small GTPases that regulate the mineralization process is crucial for the development of novel imaging techniques and therapeutic strategies counteracting pathological mineralization [5]. In the present study, using biochemical methods and microscopy techniques, we investigated novel roles of Src kinase and ROCK signaling pathways in the regulation of the mineralization of human osteosarcoma Saos-2 cells, with special emphasis on the role of AnxA6.

## 2. Results

### 2.1. Characterization of the Mineralization Process in Human Osteosarcoma Saos-2 Cells

**Figure 1:**
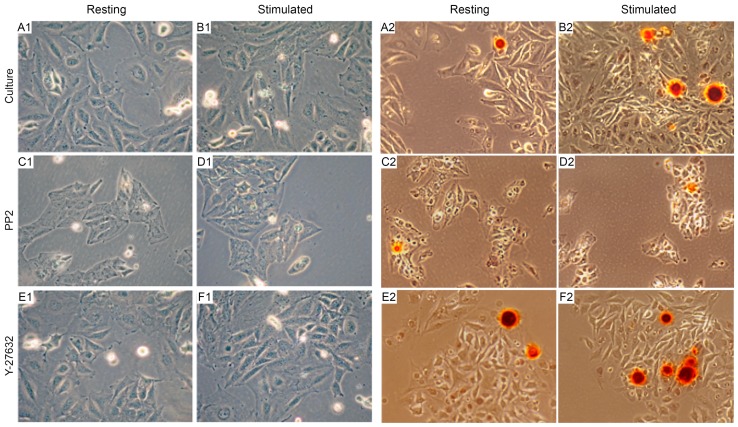
(**A1**–**F1**) Morphology of Saos-2 cells in resting conditions (**A1**,**C1**,**E1**) or after seven-day stimulation with absorbic acid (AA) and β-glycerophosphate (β-GP) (**B1**,**D1**,**F1**). Cells were either non-treated (**A1**,**B1**) or treated with inhibitors: 20 μM of 4-amino-5-(4-chlorophenyl)-7-(t-butyl)pyrazolo [3,4-d] pyrimidine (PP2) for sarcoma proto-oncogene tyrosine-protein (Src) kinases (**C1**,**D1**) or 20 μM of (R)-(+)-trans-4-(1-aminoethyl)-N-(4-pyridyl) cyclohexane carboxamide hydrochloride (Y-27632) for Rho-associated coiled-coil (ROCK) kinases (**E1**,**F1**) and observed under an Axiovert 40C light microscope (Zeiss) with phase contrast, magnification 120×. (**A2**–**F2**) Morphology of mineralizing Saos-2 cells in resting conditions (**A2**,**C2**,**E2**) or after stimulation with AA and β-GP (**B2,D2,F2**). Cells were either non-treated (**A2,B2**) or treated with inhibitors: 20 μM of PP2 for Src kinases (**C2**,**D2**) or 20 μM of Y-27632 for ROCK kinases (**E2**,**F2**). The cells were stained with AR-S and observed under an Axio Observer Z1 fluorescence microscope (Carl Zeiss, Oberkochen, Germany) with polarization-optical transmitted light differential interference contrast (PlasDIC) and red-green-blue (RGB) filters, magnification 120×.

Human osteosarcoma Saos-2 cells were incubated over seven days with 50 μg/mL of ascorbic acid (AA) and 7.5 mM of β-glycerophosphate (β-GP) (stimulated) or without these agents (resting). Both resting and stimulated cells were additionally treated with inhibitors (4-amino-5-(4-chlorophenyl)-7-(t-butyl)pyrazolo [3,4-d] pyrimidine (PP2) for Src kinase or Y-27632 for ROCK) or left untreated (culture). The addition of PP2, an inhibitor of tyrosine kinases from the Src family, impaired the adhesion of both stimulated and resting cells (Figure 1C1,D1) as compared to non-treated cells (Figure 1A1,B1). Y-27632, an inhibitor of serine-threonine kinases from the ROCK family, maintained adhesion to the surface (Figure 1E1,F1). 

To evaluate the effect of inhibitors on the adhesion of Saos-2 cells, the cell area of 10 cells from each panel—A1 to F1 of Figure 1—was measured in μm^2^ and presented as a cell area of control (Figure S1). PP2 blocked cell adhesion in 50% of the resting cells and 25% of the stimulated cells, whereas Y-27632 blocked cell adhesion in 50% of the resting and 25% of the stimulated cells in comparison to non-treated cells (Figure S1).

To evaluate the effect of inhibitors on minerals produced by Saos-2 cells, calcium salts were visualized by staining with Alizarin Red-S (AR-S), which detects calcium deposits. Stimulated cells produced many more calcium nodules than resting cells (Figure 1B2 versus Figure 1A2). The addition of PP2 decreased the ability of Saos-2 cells, as compared to control cells, to produce calcium minerals (Figure 1C2,D2 versus Figure 1A2,B2). Inhibition of the activity of ROCK by Y-27632 seems to not affect the process of mineral forming by Saos-2 when tested by less specific AR-S staining (Figure 1E2,F2 versus Figure 1A2,B2).

**Figure 2:**
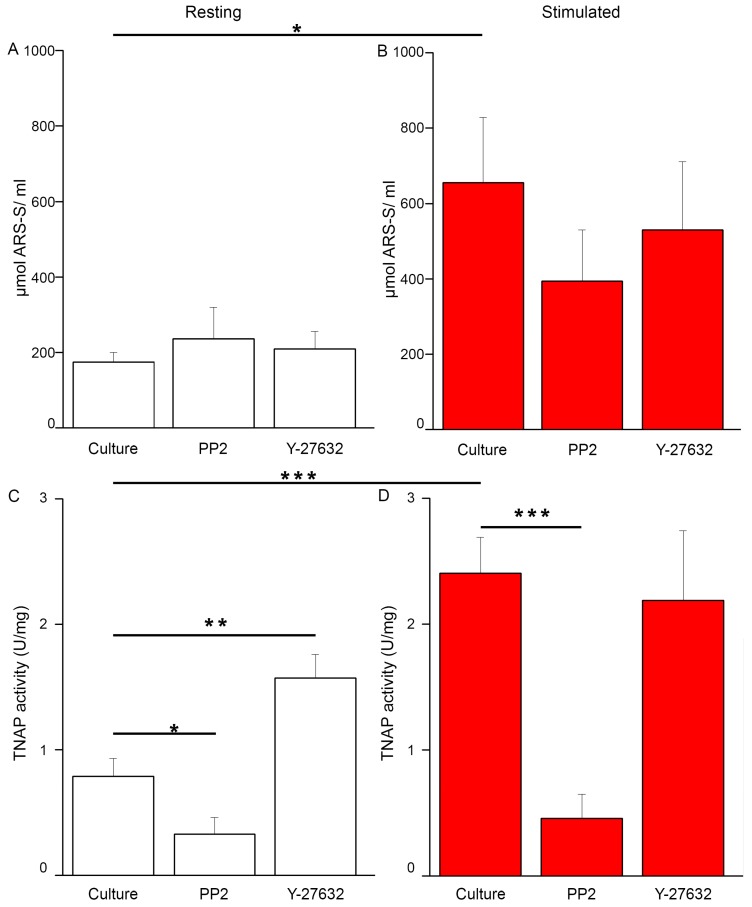
(**A**,**B**) Mineralization level of Saos-2 cells, non-treated or treated with different inhibitors, in resting conditions (**A**) or after seven-day stimulation with AA and β-GP (**B**). Both panels (**A**,**B**) are labeled uniformly: untreated cells (Culture) or cells incubated with different inhibitors: 20 μM of PP2 or 20 μM of Y-27632. Ca salts were stained with Alizarin Red-S (AR-S) and dissolved in cetylpyridinium chloride (CPC), and their content was measured spectrophotometrically at λ 562 nm, *n* = 6, * *p* < 0.05. (**C**,**D**) Tissue non-specific alkaline phosphatase (TNAP) activity in Saos-2 cells in resting conditions (**C**) or after stimulation with AA and β-GP (**D**). Cells were either non-treated or treated with different inhibitors. Both panels (**C**,**D**) are labeled uniformly: untreated cells (Culture) or cells incubated with different inhibitors: 20 μM of PP2 or 20 μM of Y-27632. TNAP activity was measured using ALP Yellow pNPP Liquid Substrate System for ELISA (Sigma, Saint Louis, MO, USA), and the absorbance was recorded spectrophotometrically at λ 405 nm, *n* = 3, * *p* < 0.05, ** *p* < 0.01, *** *p* < 0.001.

Quantitative analysis by the de-staining of calcium deposits with cetylpyridinium chloride (CPC) confirmed that stimulated cells with or without inhibitors produced many more calcium deposits than resting cells (Figure 2B versus Figure 2A). The addition of PP2 and Y-27632 to stimulated cells (Figure 2B) did not significantly decrease mineralization as compared to control cells tested by more specific CPC de-staining (Figure 2, Culture B). The inhibition of calcium nodule production by PP2 in resting and stimulated Saos-2 cells was concentration-dependent (Figure S2A), but the effect of Y-27632 was similar in both concentrations that were used (Figure S2B).

Stimulated cells had increased TNAP activity in comparison with resting cells (Figure 2D versus Figure 2C). In contrast, the addition of PP2 decreased the activity of TNAP in both resting (Figure 2C) and stimulated cells (Figure 2D) in a statistically significant way as compared to control (Figure 2C,D, Culture). The addition of Y-27632 did not affect TNAP activity in stimulated Saos-2 (Figure 2D, compare to Figure 2D, Culture). TNAP activity in Saos-2 cells that were stimulated for mineralization was modified mainly by the inhibition of Src kinase activity, but not by inhibiting ROCK kinase activity.

### 2.2. Saos-2 Cells Viability and Proliferation during Inhibition of the Mineralization Process

Our experimental conditions involving different inhibitors had no significant effects on the viability of resting or stimulated cells (Figure S3A,B). There was no discernible effect on cell cycle, and only after PP2 treatment did some cells, both resting and stimulated, became apoptotic (Figure S3C,D). Less than 25% of the experimental as well as control cells were at the G0 or G1 phase (Figure S3E,F). Almost 25% of the cells performed DNA synthesis and chromosome duplication, and only after PP2 treatment did some cells stopped proliferating (Figure S3G,H). Up to 30% of the resting and stimulated cells were in the G2 phase or performed chromosome separation, mitosis, and cell division (Figure S3I,J).

### 2.3. Protein Profile of Mineralizing Saos-2 Cells

**Figure 3:**
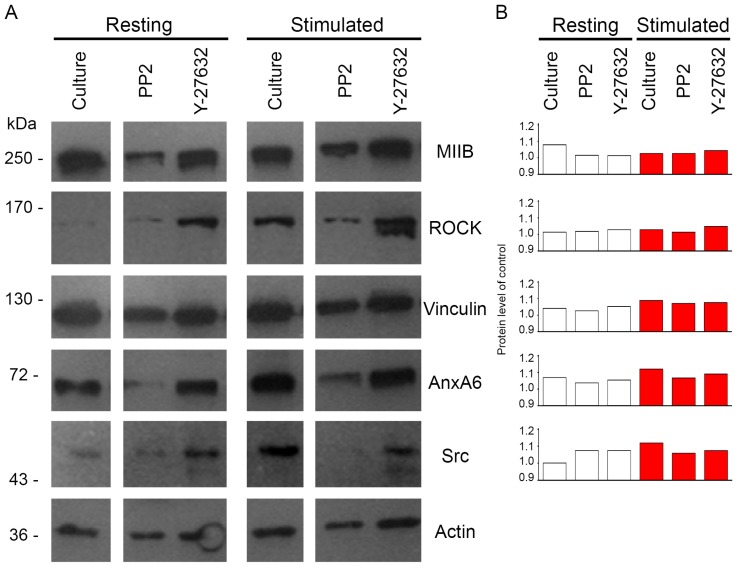
Protein profile in Saos-2 cells, non-treated (Culture) or treated with different inhibitors: 20 μM of PP2 or 20 μM of Y-27632, in resting conditions or after seven-day stimulation with AA and β-GP. Whole cell lysates were prepared in Triton Lysis Buffer (TLB). Western blot (WB) (**A**) were incubated with appropriate primary antibodies followed by secondary antibodies conjugated with horseradish peroxidase (HRP). The level of presented proteins was quantified using InGenius software (Syngene) and calculated per actin level and presented as protein level of control (**B**). Data of a typical experiment are presented.

Extracts of 5 × 10^8^ cells were homogenized in TLB buffer (0.1% Triton X-100, 0.1% β-mercaptoethanol, 1 mM of ethylenediaminetetraacetic acid (EDTA), 1 mM of EGTA, 1 μg/mL Protease Inhibitor Cocktail, 0.2 mM of phenylmethylsulfonyl fluoride (PMSF), 2 mM of NaF, 2 mM of Na_3_VO_4_, 50 mM of Tris-HCl, pH 8.0), and centrifuged. The pellets were analyzed to determine their protein profiles by Western blot (WB) (Figure 3). Molecular weights of proteins: 200 kDa may correspond to anti-non-muscle myosin IIB (MIIB), 160–150 kDa may correspond to ROCK, 120–130 kDa may correspond to vinculin, 70 kDa may correspond to AnxA6, 52–58 kDa may correspond to Src, and 40 kDa may correspond to actin (Figure 3A). The addition of Y-27632 increased ROCK content in both resting and stimulated cells as compared to control cells without any inhibitors (Figure 3B). The content of MIIB, similarly to ROCK, was altered after the treatment of cells with Y-27632, confirming the strong correlation of these proteins—that is, of the enzyme and the substrate--in vesicular structures budding from the membranes of osteoblasts. We observed a decrease in Src upon the addition of PP2 in stimulated cells as compared to control-stimulated cells (Figure 3B). The content of AnxA6, similar to that of Src, was altered after the treatment of cells with PP2, confirming the participation of these proteins in the structures of the submembraneous cytoskeleton of mineralizing Saos-2 cells. Vinculin level, similarly to Src and AnxA6, increased after stimulation for mineralization but, in opposite to these proteins, it was not significantly changed by treatment with inhibitors (Figure 3B). Actin was used as a WB marker.

### 2.4. Annexins Distribution during Stimulation of Saos-2 Cells to Mineralize

To examine the influence of AnxA6 on mineralization in the presence of inhibitors, immunocytochemical stainings were performed to detect the distribution of selected proteins in the cell.

**Figure 4:**
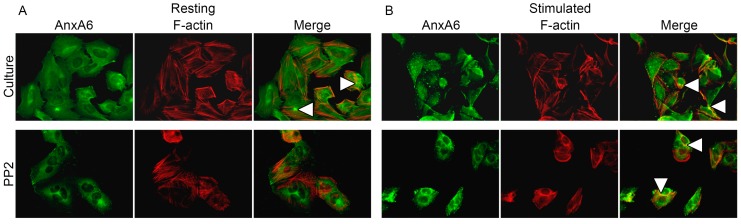
Co-localization of AnxA6 with F-actin in Saos-2 cells, non-treated (Culture) or treated with inhibitors, in resting conditions (**A**) or after seven-day stimulation with AA and β-GP (**B**). The cells were incubated with appropriate probes: mouse monoclonal anti-AnxA6 linked with goat-anti mouse Immnoglobulin G (IgG)-fluorescein isothiocyanate (FITC) and phalloidin-TexasRed recognizing F-actin and observed under an Axio Observer Z1 fluorescent microscope (Carl Zeiss, Oberkochen, Germany) with phase contrast and appropriate fluorescent filters, magnification 240×. The yellow color and arrowheads on the merge images indicate protein co-localization. Data of a typical experiment are presented.

**Figure 5:**
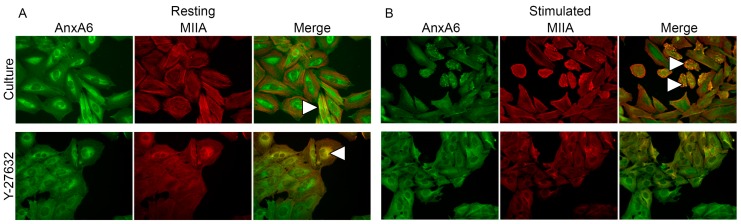
Co-localization of AnxA6 with non-muscle myosin II (MII) in Saos-2 cells, non-treated or treated with inhibitors, in resting conditions (**A**) or after seven-day stimulation with AA and β-GP (**B**). The cells were incubated with appropriate antibodies: mouse monoclonal anti-AnxA6 linked with goat-anti mouse IgG-FITC and rabbit polyclonal anti-MIIA linked with goat anti-rabbit IgG-tetramethylrhodamine (TRITC) and was observed under an Axio Observer Z1 fluorescent microscope (Carl Zeiss, Oberkochen, Germany) with phase contrast and appropriate fluorescent filters, magnification 240×. The yellow color and arrowheads on the merge images indicate protein co-localization. Data of a typical experiment are presented.

Immunocytochemical stainings specific to AnxA6 (green: fluorescein isothiocyanate (FITC)), relative to phalloidin-TexasRed from *Amonita phalloides* (F-actin) (red: phalloidin) (Figure 4), anti-non-muscle myosin IIA (MIIA) (red: tetramethylrhodamine isothiocyanate (TRITC)) (Figure 5) or vinculin (red: TRITC) (Figure S4) were performed using specific primary antibodies against these proteins followed by appropriate secondary antibodies conjugated with the indicated fluorophores. In resting Saos-2 cells, AnxA6 (Figure 4, Figure 5 and Figure S3) accumulated in cytoplasm, forming a characteristic ring in the perinuclear region. After stimulation of the cells for mineralization, AnxA6 is displaced to the inner surface of the cellular membrane (Figure 4, Figure 5 and Figure S4). F-actin (Figure 4) in resting cells was shown to be in the form of stress fibers present beneath the plasma membrane and distributed evenly throughout the cell. In stimulated cells, its presence increased in the regions of the membrane that are engaged in mineralization. The absence of any yellow color on the Merge images (resulting from the imposition of green and red fluorescent labels of the individual dyes) proves that F-actin does not co-localize with AnxA6 in mineralizing Saos-2 osteoblasts, but rather serves as a scaffold for calcium-binding proteins in vesicular structures (Figure 4, arrowheads). The distribution of vinculin (Figure S4), a protein responsible for cell adhesion to the ECM or substrate for anchoring actin filaments in the cell membrane, is similar to that of F-actin: in resting cells, it is visible as small fibers under the plasma membrane and uniformly in the whole cell. In stimulated cells, it is displaced to membrane regions involved in mineralization (Figure S4, arrowheads). Vinculin, as F-actin, also does not co-localize with AnxA6 in mineralizing osteosarcoma Saos-2 cells. The presence of PP2 (Figure 4, Figure S3) influenced cell morphology, making cells round, and thus increased the pool of AnxA6 around the nucleus that is not dependent on Src, mainly in stimulated cells. PP2 did not affect F-actin or vinculin distributions, because the structure of these fibers was preserved. Actin and AnxA6 did not co-localize in vesicles, but they did in cell-to-cell contact sites (Figure 4, arrowheads), whereas the accumulation of vinculin strongly coincided with the AnxA6 ring (Figure S4, arrowheads). MIIA (Figure 5) in resting cells was visible in the form of densely arranged fibers that, together with actin, formed the submembrane cytoskeleton of the cells. In stimulated cells, it was clearly visible that MIIA was translocated to membrane regions. This was confirmed by her tight co-localization with AnxA6 in these vesicular structures (Figure 5, arrowheads). Y-27632 (Figure 5) affected the structure of MIIA fibers in resting as well as in stimulated cells. The redistribution of AnxA6 toward the plasma membrane and co-localization with MIIA did not occur in the presence of Y-27632 in stimulated cells.

**Figure 6:**
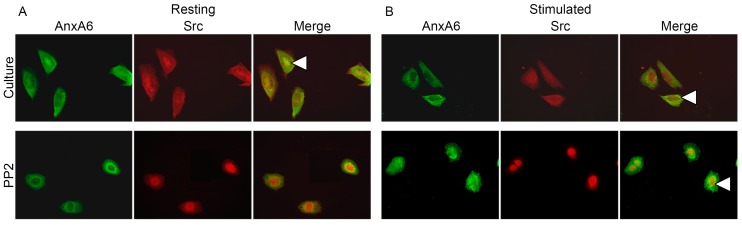
Co-localization of AnxA6 with Src in Saos-2 cells, non-treated or treated with different inhibitors, in resting conditions (**A**) or after seven-day stimulation with AA and β-GP (**B**). The cells were incubated with appropriate antibodies: mouse monoclonal anti-AnxA6 linked with goat-anti mouse IgG-FITC and rabbit polyclonal anti-Src linked with goat anti-rabbit IgG-TRITC and observed under an Axio Observer Z1 fluorescent microscope (Carl Zeiss, Oberkochen, Germany) with phase contrast and appropriate fluorescent filters, magnification 240×. The yellow color and arrowheads on the merge images indicate protein co-localization. Data of a typical experiment are presented.

**Figure 7:**
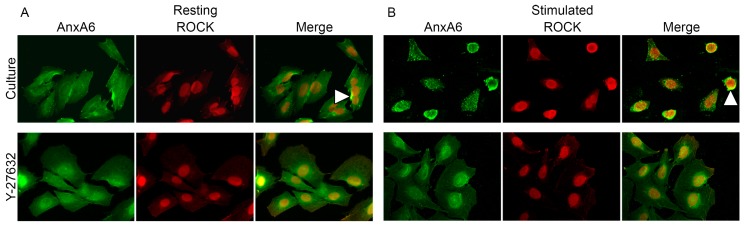
Co-localization of AnxA6 with ROCK in Saos-2 cells, non-treated or treated with different inhibitors, in resting conditions (**A**) or after seven-day stimulation with AA and β-GP (**B**). The cells were incubated with appropriate antibodies: mouse monoclonal anti-AnxA6 linked with goat-anti mouse IgG-FITC and rabbit polyclonal anti-ROCK 2 linked with goat anti-rabbit IgG-TRITC and observed under an Axio Observer Z1 fluorescent microscope (Carl Zeiss, Oberkochen, Germany) with phase contrast and appropriate fluorescent filters, magnification 240×. The yellow color and arrowheads on the merge images indicate protein co-localization. Data of a typical experiment are presented.

Immunocytochemical stainings specific to AnxA6 (green-FITC) and relative to Src (red-TRITC) (Figure 6) or ROCK (red-TRITC) (Figure 7) were performed using specific primary antibodies against these proteins followed by appropriate secondary antibodies conjugated with indicated fluorophores. In resting Saos-2 cells, AnxA6 (Figure 6 and Figure 7) accumulated in cytoplasm in the perinuclear region, whereas after the stimulation of cells for mineralization, it was redistributed to the submembrane region forming vesicles. In resting cells, Src kinase (Figure 6, arrowheads) was localized in the cytoplasm, mainly in the perinuclear region, where it co-localized with AnxA6. Upon cell stimulation, Src remained in cell cytoplasm and did not co-localize with AnxA6. The addition of PP2 did not affect AnxA6 localization in the membrane or that of Src in the cytoplasm (Figure 6). Similarly to Src kinase, in resting cells, ROCK kinase (Figure 7, arrowheads) accumulated under the nuclear envelope, where it co-localized with AnxA6, whereas after stimulation, this kinase, in contrast to Src kinase, changed its localization to the submembrane actomyosin cortex together with AnxA6 (Figure 7, arrowheads versus Figure 4 and Figure 5). Blocking the activity of ROCK kinase by Y-27632 did not affect the distribution of AnxA6 in resting cells, whereas it had a significant impact on stimulated cells, in which the redistribution of AnxA6 toward the membrane was inhibited (Figure 7). The whole pool of ROCK kinase, mainly in mineralizing cells, accumulated unspecifically in the nucleus, where it co-localized with AnxA6 (Figure 7, arrowheads).

### 2.5. Changes of Src and ROCK Kinase Activities during Mineral Formation by Saos-2 Cells

**Figure 8:**
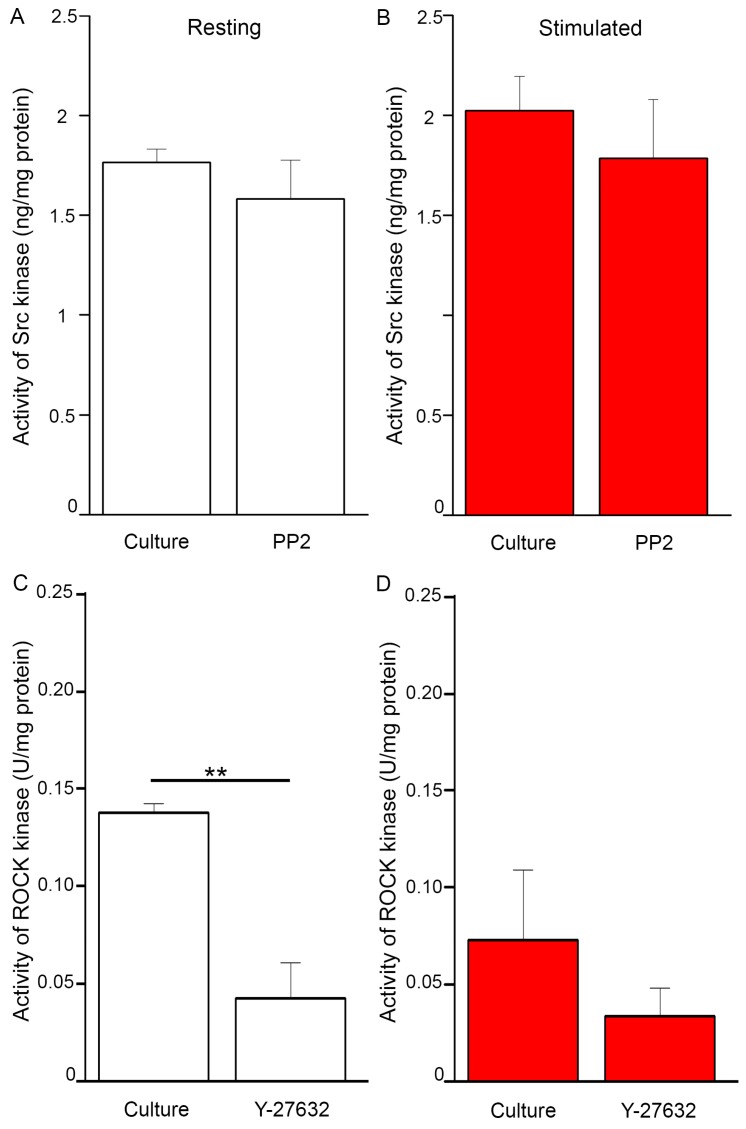
(**A**,**B**) The activity of Src kinase in Saos-2 cells, non-treated or treated with inhibitors, in resting conditions (**A**) or after stimulation with AA and β-GP (**B**). The activity was measured by a STAR Src ELISA Kit (Upstate-Millipore-Sigma, Saint Louis, MO, USA) using a SpectraMax M5e reader (Molecular Devices), Ab = λ 450 nm, (*n* = 4). (**C**,**D**) The activity of ROCK kinase in Saos-2 cells, non-treated or treated with inhibitors, in resting conditions (**C**) or after stimulation with AA and β-GP (**D**). The activity was measured with ATP, without ATP (negative control), or with ATP and Y-27623 together (positive control) by a CycLex Rho-kinase Assay Kit (CycLex Co., Ltd., Nagano, Japan) using a SpectraMax M5e reader (Molecular Devices), Ab = λ 450/540 nm, results were calculated as a difference between data obtained with ATP versus data obtained with ATP and Y-27632 together (*n* = 3), ** *p* < 0.01.

In stimulated cells, the activity of Src kinase (Figure 8B) was a little higher than in resting conditions (Figure 8A), which may support the involvement of Src in the process of mineralization. Contrary to expectations, in cells treated with PP2, the activity of this kinase, in comparison to culture without inhibitors, was only slightly reduced. Figure 8C,D indicates that the activity of ROCK was strongly dependent on the presence of ATP in the cell. The absence of ATP rendered ROCK inactive (data not shown). In stimulated cells, in the experiment with the addition of ATP (Figure 8D), ROCK activity was the lowest in cells with Y-27632. Y-27632 that was added in the presence of ATP decreased the activity of ROCK in all the experimental variants comparing to probes with ATP alone, which confirmed its potent inhibitory action against the kinase. This inhibitor decreased the activity of ROCK by almost 50% in both resting and stimulated cells (Figure 8C,D).

## 3. Discussion

Stimulated Saos-2 cells, in contrast to strongly attached resting cells, started to mineralize (Figure 1B2 versus Figure 1A2, Figure 2B versus Figure 2A), and had higher TNAP activity (Figure 2D versus Figure 2C), as we showed before [8,9,10]. Furthermore, in this work, we have shown that in such cells, Anx6 translocates from the perinuclear region of the cytoplasm toward the plasma membrane (Figure 4, Figure 5 and Figure S3). Our results from osteoblast-like cells suggested that the accumulation of AnxA6 toward the plasma membrane will contribute to the formation of MVs containing highly enriched Anx6, rendering osteoblast cells more prone to mineralization. Such a hypothesis is consistent with that suggesting that a lack of AnxA6 in chondrocytes resulted in reduced TNAP activity, the Ca^2+^ to P_i_ ratio, and the inability of cells to form apatite crystals in vitro [23]. The application of different inhibitors that are selective for kinases from Src and ROCK families affected the morphology of mineralizing cells and changed their abilities to adhere, proliferate, and produce minerals. We used two inhibitors to probe the effects of Src and ROCK on mineralization: PP2, as an inhibitor of Src kinase activity, and Y-27632 as an inhibitor of ROCK kinase activity. The production of calcium nodules by resting and stimulated Saos-2 cells was differently modulated by inhibitors depending on their concentrations (Figure S2). Our experimental conditions i.e., the presence of inhibitors, had no significant effects on cell viability and the cell cycle of resting or stimulated cells, but actomyosin cortex-regulated mineralization was probably accompanied by osteoblast apoptosis (Figure S3) [12]. The results obtained by us are in agreement with data that indicate the different regulation of mineralization by kinases from the Src and ROCK families [5]. (1) The addition of PP2 inhibited the mineralization process as probed by AR-S (Figure 1), TNAP activity (Figure 2), and Src, ROCK, and AnxA6 protein levels (Figure 3). A significant pool of Src and AnxA6 co-localized in the cytoplasm (Figure 6), suggesting that AnxA6 did not migrate toward the plasma membrane effectively enough to ensure proper mineralization. In contrast, the addition of Y-27632 did not significantly impair the mineralization process as probed by AR-S (Figure 1), TNAP activity (Figure 2), and the Src, ROCK, and AnxA6 protein levels (Figure 3). In that case, AnxA6 remained in the cytoplasm (Figure 7) where it co-localized with ROCK (Figure 7). AnxA6 distribution during MVs release by osteoblasts strongly correlated with actin cytoskeleton polymerization (Figure 4) and the formation of the MII ring (Figure 5), but less with alterations in focal adhesion (Figure S4), confirming the data obtained earlier [4,21,22]. We observed slight changes in Src kinase activity, whereas ROCK activity was very sensitive to fluctuations in mineral deposition after cell treatment with a specific inhibitor (Figure 8). These observations are reminiscent of a report showing that in vitro, one of the Src kinases, Fyn, mediated actin cytoskeleton remodeling via the promotion of ROCK activation [24]. Recently, the roles of the Src and ROCK pathways in the regulation of various aspects of tumor cells functions, such as progression and metastasis, have been characterized [25]. The results provide insights into mechanisms underlying some events mediated by downstream caveolin-1 pathways, including FAK/Src and ROCK/p-myosin light chain (MLC), which are involved in the reorganization of the cytoskeleton, focal adhesion dynamics, cancer cell motility, and adhesion.

Taken together, the findings that we describe in this report provide clear-cut evidence that the inhibitor of Src kinase elicited an impairment of the mineralization process, indicating that Src kinase is essential for proper mineralization. We conclude that in human osteosarcoma Saos-2 cells, Src kinase is activated during early steps of mineral formation, which leads to the reorganization of the actin cytoskeleton, whereas ROCK is engaged in MII rearrangement and vesicle release [6].

## 4. Materials and Methods

### 4.1. Cell Culture and Treatment

Human osteosarcoma Saos-2 cells (ATCC HTB-85) were cultured in McCoy’s 5A medium with 1.5 mM of L-glutamine (ATCC) supplemented with 100 U/mL of penicillin, 100 U/mL of streptomycin (Sigma, Saint Louis, MO, USA), and 15% fetal bovine serum (*v:v*, FBS, Gibco-Fisher Sci., Waltham, MA, USA). The cells were grown at 37 °C in atmosphere of 5% CO_2_.

Cells were stimulated for mineralization one day after cell passage and attachment by treatment with 50 μg/mL of ascorbic acid (AA, Sigma, Saint Louis, MO, USA) and 7.5 mM of β-glycerophosphate (β-GP, Sigma, Saint Louis, MO, USA) for 7 days [26]. The mineralization process was modulated by the addition of either 20 μM of 4-amino-5-(4-chlorop*h*enyl)-7-(t-butyl)pyrazolo[3,4-d]pyrimidine (PP2, an inhibitor of Src, Calbiochem-Sigma, Saint Louis, MO, USA), or 20 μM of (R)-(+)-trans-4-(1-aminoethyl)-N-(4-Pyridyl)cyklohexane carboxamide dihydrochloride monohydrate (Y-27632, an inhibitor of ROCK, Sigma, Saint Louis, MO, USA) for 7 days starting 4 h after the addition of AA and β-GP. The final concentration of DMSO as a solvent for the inhibitor solutions in cultured medium did not exceed 0.1% (*v:v*). Cell cultures were photographed under an inverted Axiovert 40C light microscope (Carl Zeiss, Oberkochen, Germany) using transmitted light and phase contrast. For adhesion quantitation, the cell area of 10 cells from each image was measured in μm^2^ using Image J bundled with 64-bit Java 2.8.0_112 software and presented as a cell area of control.

The resting and stimulated cells were analyzed for calcium mineral, TNAP activity, cell lysis, and the identification of a certain protein by using SDS-PAGE and immunoblot, immunochemistry and fluorescent microscopy, Src activity, ROCK activity, cell viability, and cell cycle.

### 4.2. Calcium Mineral Detection

The presence of calcium deposits in cells treated with different inhibitors was detected by staining with Alizarin Red-S (AR-S, Sigma, Saint Louis, MO, USA) [27]. Cells were washed with phosphate buffer saline and incubated with 0.5% (*w:v*) AR-S in Phosphate-Buffered Saline (PBS) pH 5.0 for 30 min at room temperature. Then, cultures were washed three times with PBS to remove free calcium ions, and stained calcium deposits attached to the cultures were photographed under an inverted Axio Observer Z1 fluorescent microscope (Carl Zeiss, Oberkochen, Germany) using transmitted light, PlasDIC contrast, and red/green/blue (RGB) filters. The analysis of calcium salts levels in the cells was done by de-staining with cetylpyridinium chloride (CPC, Sigma, Saint Louis, MO, USA) [28]. Cell cultures were incubated with 10% (*w:v*) CPC in PBS, pH 7.0, for 30 min at room temperature. The obtained solution was centrifuged at 130× *g* for 1 min at room temperature (MPW-350R, MPW Medical Instrument). The collected supernatant was used for measurements of absorbance at 562 nm using a BioMate3 spectrophotometer (Thermo Electron Co., Beverly, MA, USA). [Ca^2+^] was calculated and referenced to the standard curve of AR-S as described earlier [29].

### 4.3. TNAP Activity Assay

10^8^ cells treated with different inhibitors, either resting or stimulated for 7 days, were digested with collagenase as described in [30]. Medium from cellular cultures was collected while cells were washed with PBS and incubated with crude collagenase (500 U/mL, type IA; Sigma, Saint Louis, MO, USA) in a solution of 0.25 M of sucrose, 0.12 M of NaCl, 0.01 M of KCl, and 0.02 M of Tris-HCl buffer, pH 7.45, at 37 °C for 3 h. Then, cells were mechanically scraped, passed 10 times through a 0.5 × 16 syringe, sonicated twice on ice for 10 s at 20% power of a S-250D digital sonifier (Branson Ultrasonic S.A., Danbury, CT, USA), and centrifuged at 500× *g* for 5 min at 4 °C (MPW-350R, MPW Medical Instrument, Warsaw, Poland). The pellet was suspended in 500 μL of Hank’s balanced salt solution (HBSS, 5.4 mM of KCl, 0.3 mM of Na_2_HPO_4_, 0.6 mM of KH_2_PO_4_, 0.6 mM of MgSO_4_, 137 mM of NaCl, 5.6mM of D-glucose, 2.38 mM of NaHCO_3_, pH 7.4). The collected supernatant was analyzed for protein concentration, using the Micro BCA Reagent (Pierce-Thermo Fisher Sci., Waltham, MA, USA), and absorbance was measured at 562 nm in a BioMate3 spectrophotometer (Thermo Electronics Co., Beverly, MA, USA). The collected supernatant was also analyzed for TNAP activity, using an ALP Yellow pNPP (paranitrophenylphosphate) Liquid Substrate System for ELISA (Sigma, Saint Louis, MO, USA). The reaction was initiated with the addition of 10-μL (0.5 μg of protein) aliquots of the enzyme to 96-well plates containing 200 μL of pNPP as substrate. The plates were incubated at 37 °C for 5 min, and the absorbance was measured at 405 nm for 1 h with 15 s intervals using a Spectra Max M5e multi-detection reader (Molecular Devices). Reaction was terminated using 50 μL of 3 M of NaOH. TNAP activity was expressed in U/mg protein, where 1 U = 1 μmol pNPP hydrolyzed per min and visualized by Origin 7.5 software (OriginLab Co., Northampton, MA, USA).

### 4.4. Cell Lysis, SDS-PAGE, and Immunoblot Analysis

10^8^ cells treated with different inhibitors, either resting or stimulated for 7 days, were lysed in RIPA (150 mM of NaCl, 0.25% deoxycholic acid, 1% NP-40, 1 mM of EDTA, 1 μg/ml of Protease Inhibitor Cocktail (Sigma, Saint Louis, MO, USA), 1 mM of PMSF, 50 mM of Tris-HCl, pH 7.4) or TLB (0.1% Triton X-100, 0.1% β-mercaptoethanol, 1 mM of EDTA, 1 mM EGTA, 1 μg/mL Protease Inhibitor Cocktail (Sigma), 0.2 mM PMSF, 2 mM NaF, 2 mM Na_3_VO_4_, 50 mM Tris-HCl, pH 8.0) buffers according to Src or ROCK activity assays protocols, respectively. Medium from cell cultures was removed, while cells were washed with PBS and incubated with 1 ml of lysis buffers at 4 °C for 15 min. Then, cells were mechanically scraped, vortexed for 10 sec, sonicated on ice for 10 sec at 20% power of a S-250D digital sonifier (Branson Ultrasonic S.A., Danbury, CT, USA), and centrifuged at 13,000× *g* for 10 min at 4 °C (MPW-350R, MPW Medical Instrument, Warsaw, Poland). The collected supernatant was analyzed for protein concentration using the Bradford Reagent (Bio-Rad, Hercules, CA, USA), and absorbance was measured at 595 nm in a BioMate3 spectrophotometer (Thermo Electron Co., Beverly, MA, USA). The supernatant was divided into 100 μL samples and stored at −80 °C.

Proteins of cell lysates were separated on 10% (*w:v*) SDS-PAGE [31] and then electrotransferred (Mini-ProteanIITM Kit, Bio-Rad, Hercules, CA, USA) onto nitrocellulose membranes (HybondTM-ECLTM, Amersham Biosciences, Amersham, Buckinghamshire, UK) according to Towbin et al. [32]. Nitrocellulose membranes were blocked with 5% (*w:v*) milk in TBS for 1 h at room temperature. Then, the membranes were incubated with mouse monoclonal anti-annexin A6 (AnxA6; 1:1000, *v:v*; Transduction Laboratories), rabbit polyclonal anti-vinculin (vinculin; 1:500, *v:v*; Sigma, Saint Louis, MO, USA), rabbit polyclonal anti-non-muscle myosin IIB (MIIB; 1:500, v:v; Sigma, Saint Louis, MO, USA), rabbit polyclonal anti-Src kinase (Src; 1:500, *v:v*; Sigma, Saint Louis, MO, USA) or rabbit polyclonal anti-ROCK 2 kinase (ROCK; 1:1000, v:v; Sigma) or mouse monoclonal anti-actin 1 (Actin; 1:2000) primary antibodies diluted in TBS supplemented with 0.05% (*v:v*) Tween-20 (TTBS) containing 3% (*w:v*) milk at 4 °C overnight. Nitrocellulose membranes were washed several times with TTBS and incubated with sheep anti-mouse or anti-rabbit IgG secondary antibodies conjugated with horseradish peroxidase (HRP, 1:5000; both from Amersham Biosciences, Amersham, Buckinghamshire, UK) and diluted in 3% (*w:v*) milk in TTBS for 2 h at room temperature. Finally, the membranes were washed several times with TTBS, and immunoreactive protein bands were visualized on MXB X-ray films (Kodak) using ECL reagents according to the manufacturer’s instructions (Amersham Biosciences, Amersham, Buckinghamshire, UK). Then, the films were analyzed densitometricaly using InGenius software (Syngene, Cambridge, UK).

### 4.5. Immunochemistry and Fluorescent Microscopy

10^5^ cells were cultured in culture medium on cover slips overnight at 37 °C in 5% CO_2_ humidified atmosphere. Next-day stimulators (50 μg/mL of AA and 7.5 mM of β-GP), and 4-h later inhibitors (either 20 μM PP2, or 20 μM Y-27632) were added to the appropriate variants of cell cultures for 7 days. Then, cells were washed with Physiological Desensitization (PD) buffer (125 mM of NaCl, 5 mM of KCl, 10 mM of NaHCO_3_, 1 mM of KH_2_PO_4_, 10 mM of glucose, 20 mM of 2-[4-(2-hydroxyethyl)piperazin-1-yl]ethanesulfonic acid (HEPES), pH 6.9) and fixed with 3% (*w:v*) paraformaldehyde in PD buffer (20 min, room temperature) [8]. Fixed cells were incubated in 50 mM of NH_4_Cl in PD buffer (10 min, room temperature), washed with PD buffer, and then permeabilized with 0.08% (*v:v*) Triton X-100 in PD buffer (5 min, 4 °C). After additional washing with Tris-buffered saline (TBS; 100 mM of NaCl, 10 mM of Tris-HCl, pH 7.5), cells were incubated with a blocking solution, 5% (*v:v*) FBS in TBS for 45 min at room temperature. Then, cells were incubated with mouse monoclonal anti-annexin A6 (AnxA6; 1:100, *v:v*; Transduction Laboratories), rabbit polyclonal anti-vinculin (vinculin; 1:100, *v:v*; Sigma), rabbit polyclonal anti-non-muscle myosin IIA (MIIA; 1:100, *v:v*; Sigma), rabbit polyclonal anti-Src kinase (Src; 1:100, *v:v*; Sigma), or rabbit polyclonal anti-ROCK 2 kinase (ROCK; 1:100, *v:v*; Sigma) primary antibodies diluted in TTBS containing 0.5% (*w:v*) FBS. After 1 h of incubation at room temperature, the cells were washed in TBS and incubated for 1 h at room temperature with goat anti-mouse IgG-fluorescein isothiocyanate (FITC; 1:200, *v:v*) or goat anti-rabbit IgG-tetramethylrhodamine isothiocyanate (TRITC; 1:200, *v:v*; both from Sigma) secondary antibodies or with Phalloidin-TexasRed from *Amonita phalloides* (F-actin; 1:1500, *v:v*; Fluka-Sigma, Saint Louis, MO, USA) diluted in TTBS containing 0.5% (*w:v*) FBS. After washing with TBS and deionized water, the samples were mounted in 0.6% (*v:v*) Moviol 4-88 (Calbiochem-Sigma, Saint Louis, MO, USA) supplemented with 2.5% (*w:v*) DABCO (Sigma). Samples were stored at 4 °C overnight and then photographed under an Axio Observer Z1 fluorescent microscope (Carl Zeiss, Oberkochen, Germany) using reflected light, phase contrast, and appropriate fluorescent filters.

### 4.6. Src Activity Assay

Src kinase activity was measured using a STAR (Signal Transduction Assay Reaction) Src ELISA Kit (Upstate-Millipore-Sigma, Saint Louis, MO, USA), which detects phosphorylation at Tyr-418. 10^8^ cells treated with different inhibitors, either resting or stimulated for seven days, were lysed in Radioimmunoprecipitation assay (RIPA) buffer. Then, 100 μl of cell lysate in RIPA buffer was added to the wells of a 96-well plate pre-coated with anti-Src antibody and incubated overnight at 4 °C with shaking using a GENIUS 3 vortex (IKA). Then, samples were washed four times with 200 μL of ELISA wash buffer (liquids were removed by inverting plates and blotting on towel) and incubated with 100 μL of rabbit anti-Src antibody for 1 h at room temperature with shaking. Then, samples were washed four times with 200 μL of ELISA wash buffer and incubated with 100 μL of anti-rabbit IgG conjugated with HRP for 45 min at room temperature with shaking. Then, samples were washed four times with 200 μL of ELISA wash buffer and incubated 30 min at room temperature in darkness with 100 μL of tetramethylbenzidine (TMB) substrate solution. At the end, 100 μL of stop solution was added, and absorbance at 450 nm was measured within 1 h after stopping the reaction using a Spectra Max M5e multi-detection reader (Molecular Devices, San Jose, CA, USA). Src activity was calculated using protein from Src standards and visualized by Origin 7.5 software (Origin Co., Krakow, Poland).

### 4.7. ROCK Activity Assay

ROCK kinase activity was measured using a CycLex Rho-kinase Assay Kit (CycLex Co., Ltd., Nagano, Japan), which detects MBS protein phosphorylation on Thr-696 in the presence of ATP. 10^8^ cells treated with different inhibitors, either resting or stimulated for 7 days, were lysed in TLB buffer. Then, 10 μL of cell lysate in TLB buffer and 90 μL of kinase reaction buffer containing ATP were added to the wells of a 96-well plate coated with the C-terminal fragment (645-880) of the myosin-binding subunit (MBS) protein and incubated for 30 min at 30 °C in a KBC-250 oven (Wad-med, Pruszkow, Poland). Then, samples were washed five times with 200 μL of Tween washing buffer and incubated with 100 μL of AF20 (anti-phospho-MBS T696) antibody conjugated with HRP for 1 h at room temperature. Then, samples were washed five times with 200 μL of Tween washing buffer, and 100 μL of TMB substrate was added and incubated 15 min at room temperature in darkness. At the end, 100 μL of stop solution was added, and dual absorbance at 450/540 nm within 30 min after stopping the reaction was measured using a Spectra Max M5e multi-detection reader (Molecular Devices, San Jose, CA, USA). ROCK activity was calculated as U/mg protein, where 1 U = amount of ROCK transferring 1 nmol of P on MBS per min at 30 °C.

### 4.8. Cell Viability and Cell Cycle Analysis by Flow Cytometry

For cell viability analysis [33], 10^6^ cells were incubated in an appropriate buffer (resting or stimulated, control culture or with inhibitors). Afterwards, the cells were washed in the PD medium, and then suspended in 2 mL of propidium iodide (PI) solution (50 μg/mL PI in PD), incubated for 5 min at room temperature, and used directly for a flow cytometry analysis of PI fluorescence.

For cell cycle analysis [34], 3 × 10^6^ cells were incubated in an appropriate buffer (resting or stimulated, control culture or with inhibitors). Afterwards, the cells were centrifuged at 2500× *g* for 5 min, and the pellet was suspended in 1 mL of PBS. The cell suspension was transferred into 2.5 ml of absolute EtOH (final concentration approx. 70%) and fixed overnight at –20 °C. Then, the cells were centrifuged and suspended in 500 μL of PI solution for 40 min at 37 °C. After that, 3 ml of PBS was added, and the cells were centrifuged. The obtained supernatant was discarded, while the pellet was resuspended in 1 ml of PBS for flow analysis of PI fluorescence. A FACSCalibur flow cytometer (Becton-Dickinson, Franklin Lakes, NJ, USA) equipped with an argon laser emitting at 488 nm was used. In both PI staining methods, PI fluorescence was monitored by the FL2 (470 V linear) emission channel [35]. The number of cells (%) was counted using the CellQuest software (Becton-Dickinson, Franklin Lakes, NJ, USA) from 10^4^ cells per each experimental set.

### 4.9. Statistical Analysis

All the values are reported as mean ± SD. Data were analyzed by one-way ANOVA, and postdoc analyses were performed using the Tukey method with the aid of Daniel’s XL Toolbox add-in for Excel, version 6.60, by Daniel Kraus, Wurzburg, Germany [36]. Statistical significance was described as * *p* < 0.05, ** *p* < 0.01 and *** *p* < 0.001.

## Supplementary Materials

The following are available online at https://www.mdpi.com/1422-0067/20/12/2872/s1.

## Abbreviations

AA	ascorbic acid
ALP	alkaline phosphatase
AnxA	vertebrate annexin
AR-S	Alizarin Red-S
BSP	bone sialoprotein
ECM	extracellular matrix
β-GP	β-glycerophosphate
CPC	cetylpyridinium chloride
FAK	focal adhesion kinase
FN	fibronectin
MII	myosin II
MVs	matrix vesicles
P_i_	inorganic phosphate
pNPP	paranitrophenyl phosphate
PP2	4-amino-5-(4-chlorophenyl)-7-(t-butyl)pyrazolo [3,4-d] pyrimidine
PP_i_	inorganic pyrophosphate
ROCK	Rho-associated coiled-coil kinase
Src	sarcoma proto-oncogene tyrosine-protein kinase
TNAP	tissue non-specific alkaline phosphatase
Y-27632	(R)-(+)-trans-4-(1-aminoethyl)-N-(4-pyridyl) cyclohexane carboxamide dihydrochloride
