# Supplementary material for: Src and ROCK Kinases Differentially Regulate Mineralization of Human Osteosarcoma Saos-2 Cells
Source: Int J Mol Sci. 2019 Jun 12;20(12):2872. doi: 10.3390/ijms20122872 (PMC6628028; doi:10.3390/ijms20122872)
Supplement: Supplementary file 1 [file ijms-20-02872-s001.pdf]

Supplementary figures

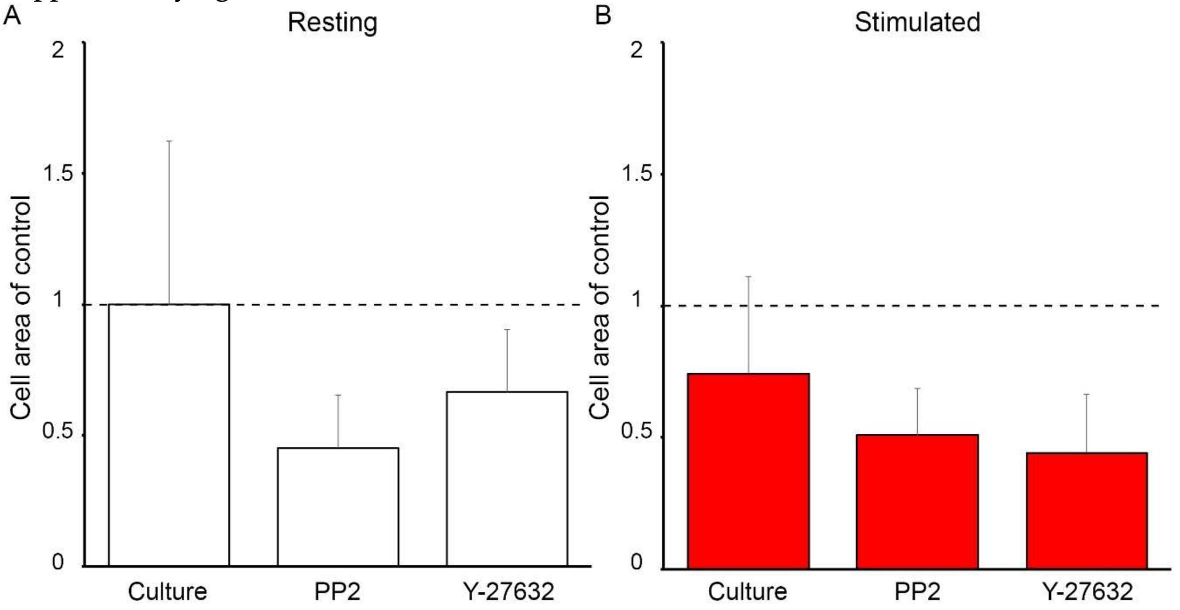

Figure S1. Adhesion of mineralizing Saos-2 cells in resting conditions (A) or after 7-day stimulation with AA and β-GP (B). The cell area of 10 cells from each panel A1-F1 of Fig. 1 was measured in μm^2 using Image J bundled with 64-bit Java 2.8.0_112 software and presented as the percentage of the area of control cells.

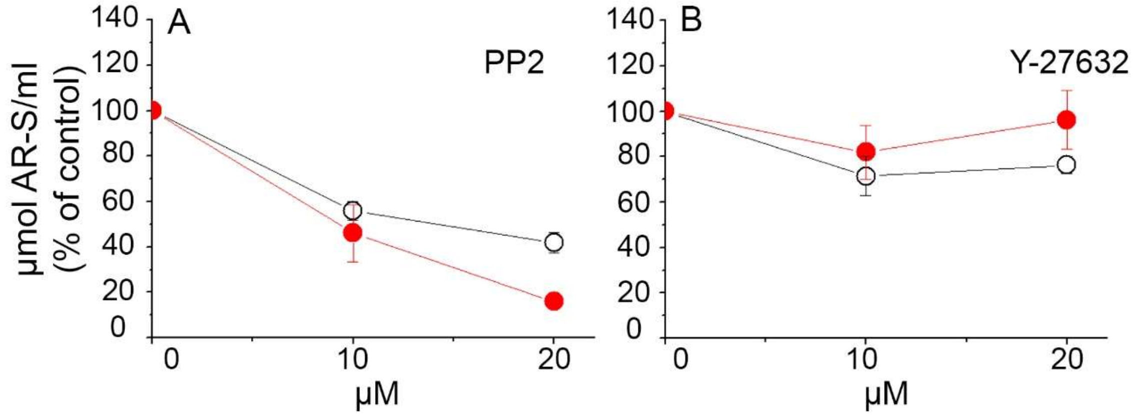

Figure S2. Concentration-dependent inhibition of mineralization of Saos-2 cells in resting conditions (white circle) or after 7-day stimulation with AA and β-GP (red circle). Cells were either non-treated or treated with different inhibitors: PP2 (A) or Y-27632 (B). Ca salts were stained with AR-S and dissolved in CPC and their content was measured spectrophotometrically at λ 562 nm as % of control, n=4-6.

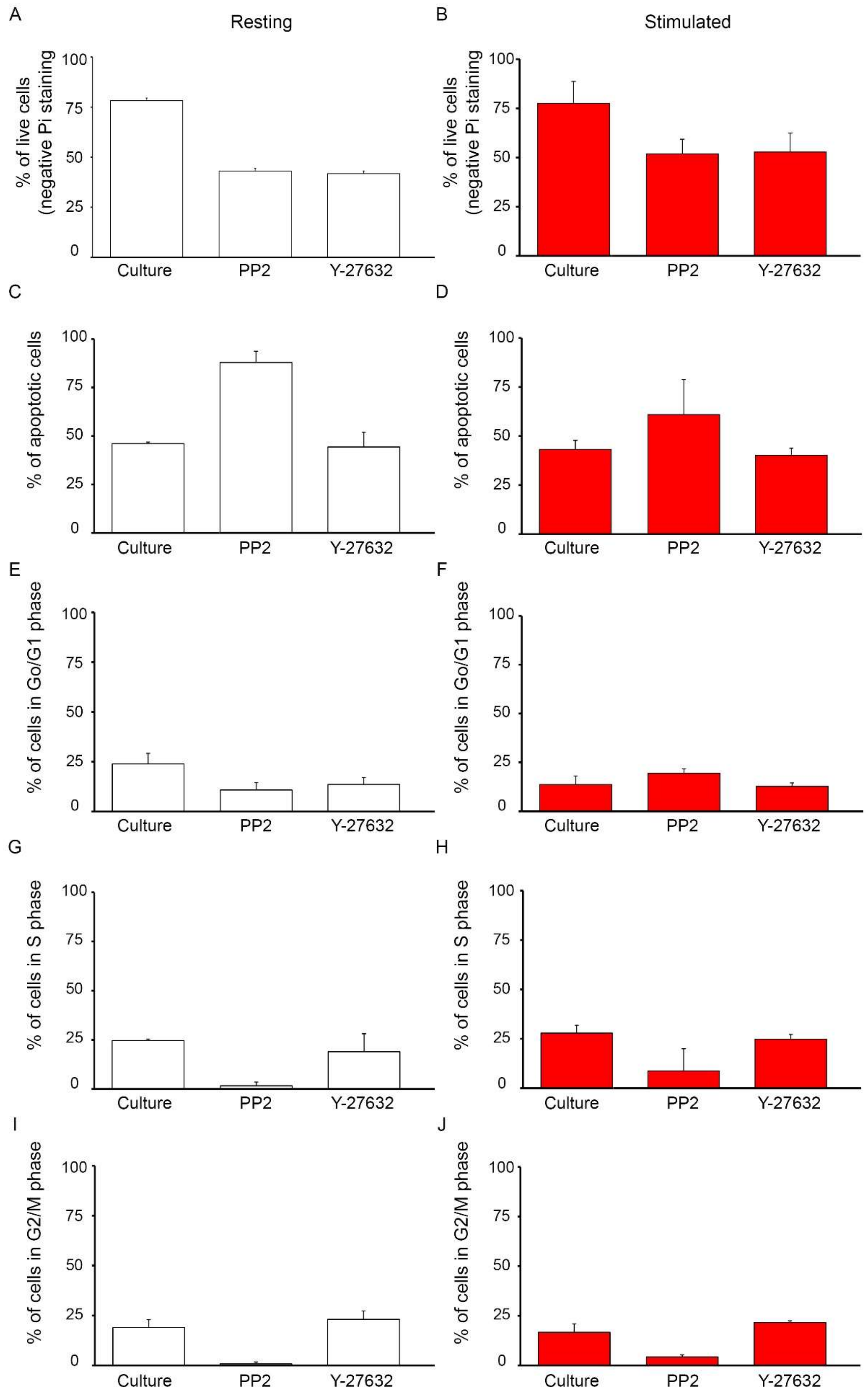

Figure S3. Saos-2 cells viability and cell cycle in resting conditions (A, C, E, G, I) or after 7-day stimulation with AA and β-GP (B, D, F, H, J). Cells were either non-treated or treated with inhibitors. All panels (A-J) are labeled: untreated cells (Culture) or cells incubated with different inhibitors: 20 μM PP2 or 20 μM Y-27632. To identify live cells (A, B), 10^6 cells were washed, stained by PI and

analyzed directly by means of flow cytometry to determine cell viability, (n=5). For cell cycle analysis (C-J), 3 x 106 cells were fixed, stained by PI, centrifuged, suspended in PBS and used for flow analysis of apoptotic cells (C, D), interphase cells (E, F), DNA synthesis (G, H) and dividing cells (I, J), (n=5).

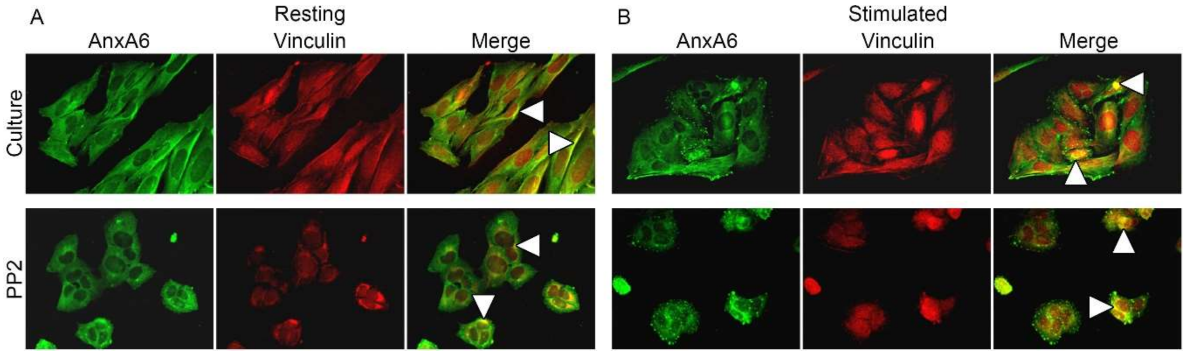

Figure S4. Co-localization of AnxA6 with vinculin in Saos-2 cells, non-treated or treated with inhibitors, in resting conditions (A) or after 7-day stimulation with AA and β-GP (B). The cells were incubated with appropriate antibodies: mouse monoclonal anti-AnxA6 linked with goat-anti mouse IgG-FITC and rabbit polyclonal anti-vinculin linked with goat anti-rabbit IgG-TRITC and observed under an Axio Observer.Z1 fluorescent microscope (Zeiss) with Phase contrast and appropriate fluorescent filters, magnification 240 x. Yellow color and arrowheads on merge images indicate protein co-localization. Data of a typical experiment are presented.
